# Anatomy

**Published:** 1836-04

**Authors:** 


### PART THIRD.

### Selections from Foreign Journals.

### ANATOMY.

*On the Structure of Bones*. By MICHELE MEDICI, M.D., Professor in the University of Bologna.

[As we are not in possession of the original work, (the Transactions of the Academy of Sciences of Bologna), in which this Essay has appeared, we must request our readers to accept so much of its contents as we have been enabled to glean from the notice of it in the Annali di Medicina. In our first number we gave a transcript of the researches of W. and F. Arnold into the structure of cartilage and bone, but not having had an opportunity of verifying either their opinions or those of Medici, we cannot point out how far these different views of the same structures are reconcileable with each other. But as the observations of the Arnolds were made with the assistance of a microscope of considerable power, while those of Medici were made by the eye alone, it is probable that any discrepancies in the descriptions of these authors may be attributable to the different magnitudes of the portions of bone examined.]

Scarpa having, by the experiments and reasoning detailed in his work, De Penit. Oss. Struct., succeeded in overthrowing (at least in Italy) the commonly received opinions of the ancient anatomists, that the bones are formed of laminæ and fibres, and having established his own theory, that they consist in every period of life of a tissue always reticulated, cellular, and alveolar, such as is observed in the bones of the fœtus; our author considers that he cannot follow a course better calculated to support his own views, than by controverting those of his great predecessor by going through the same experiments, and adducing his arguments from the same sources. Following therefore the example of Scarpa, he examines the bones in their natural state, when altered by disease, and when affected by chemical reagents, or, in the words of the author, synthetically, pathologically, and analytically.

As the bones of an adult animal present to our view neither the laminæ nor the distinct fibres of which they are composed, but only certain more solid points here and there, with cells, channels, fissures, and interlacings of every form, and as it is known that the bones of the fœtus and infant are formed of a soft cellular, alveolar tissue; so it is believed by many that the same texture is preserved through the whole of life, and that the super-addition of calcareous matter only performs the office of solidifying and strengthening the cells sufficiently to resist the action of the muscles. Such a structure is certainly seen in the internal medullary portion of the cylindrical bones, but it is scarcely observed in the broad and flat bones, and not at all in the exterior portion of the long bones, which plainly consists of interwoven laminæ and fibres. In support of this opinion the author gives fourteen drawings of the fibrous and laminated structure both of flat and cylindrical bones, perfectly distinct from the primitive cellular and reticulated appearance observed in the bones of the fœtus.

In arguing from the pathological states of the bones in caries, exostosis, and periostitis, the author does not allow, with Scarpa, that these diseases merely exhibit the bone in its original form, but deprived of its calcareous matter; but asserts that they are irregular, abnormal, and varying conditions, arising from the action of morbid processes developed in the bones themselves, and common to them with all other parts of the living body. In support of this opinion he refers to many of Scarpa's plates, in which it is quite impossible, in its altered state, to trace the primitive form of the bone; particularly in one instance, where in various preparations of the same bone its structure is obviously different. And in proof of the fibrous structure of the long bones, he instances a case of caries of the tibia of a dancing girl, where the lower end of the bone had the appearance of "combed hair."

The results of Dr. Medici's analytical experiments will be best given in his own words: "I immersed a longitudinal portion of the thigh bone in diluted muriatic acid, when, after it had become softened to a certain degree, it exhibited a fibrous texture not only on its external and internal surfaces, but also throughout its whole thickness. Having continued the maceration some time longer, the bone was so far decomposed that some flocculi or bundles of bony fibres began to separate from it; when transferred into alcohol, I observed with much pleasure these bundles fluctuating within the clear liquor whenever it was agitated. Some of them having become detached, fell to the bottom of the vessel, where they remained dry and adherent after the liquor was poured off. Examined as they lay, the fibres appeared of a yellow colour and were perfectly distinct to the eye." The direction of the bony fibres was next examined, and was ascertained by numerous experiments to be longitudinal, although Medici could not determine whether they were continuous through the whole length of the bone. They are of a cylindrical shape, and sometimes rather conical; which depends upon the varying thickness of the bone. During maceration they are translucent like little worms; but when dry they are opaque, whitish, and elastic. They are usually parallel to each other, but sometimes they separate and take an oblique course, or are even bifurcated and branched and run into each other by means of oblique or transverse bony threads. In the flat bones the laminated and fibrous structure is much more evident. The author having macerated some pieces of the parietal and occipital bones, discovered in them a distinct superposition of laminæ like the leaves of a book. He observed the same in the internal table of the same bones, which was only interrupted by the impressions of the meningeal artery; moreover he asserts that he has seen certain "filaments or beards," appendages to the osseous fibres, rising up from the inferior table, meeting the upper, and appearing to be compressed or flattened by a force from above. They are of various shapes and lengths, sometimes formed of a single filament, sometimes bifurcated, cruciform, and branched, and so moveable in the macerated bone that they are easily raised up on the point of a needle. From certain parts of the bone they are entirely absent, as between the first and second, and between the second and third plates. As they penetrate towards the centre they increase in number, so that the bone at this part appears to be composed of an innumerable congeries of fibres, forming a downy substance and throwing out points to the circumference, which give it a dentated edge. The filaments are not observable on the external surface of the bones, but only after the removal of two or three of the laminæ. They are far more distinct where they terminate in the squamous suture of the parietal bone. From hence it would appear that there is a notable difference between the cranial and the other bones; as in the latter, the filaments form a part of the laminæ, whereas in the former they separate the laminæ completely without adhering to them.

The same results are obtained from experiments on the bones of animals; a similar disposition of the osseous fibres and laminæ interlacing with each other is very evident, both in the long and flat bones.—*Annali di Medicina, Gennajo,* 1835.

---

### *Of the supposed Origin of the Posterior Root of the first Cervical from the eleventh Cerebral (Accessory) Nerve.* By Fr. Arnold.

J. Muller (in his *Archiv.* 1835, No. 1. p. 12,) claims to be the first who noticed that the accessory nerve sometimes gives off the posterior root of the first cervical, forming a little ganglion upon it within the dura mater; and adds, that "such instances prove the incorrectness of the views of Scarpa, Arnold, and Bischoff, according to which the accessory nerve is a motor only, and gives some motor fibres to the vagus; which, on account of its very distinct ganglion, should otherwise be deemed exclusively a nerve of sensation."

A reference to some writers, both of old and modern date, will prove that the case observed by Müller is by no means a new discovery, and has been better examined by earlier anatomists than by himself.

Huber (*De Medulla Spinali*, p. 13,) remarks, "Occasionally, the filaments that usually form the posterior root of the first cervical nerve are sent to the accessory; in which case the latter gives off, either in the same situation (but without any continuity with them,) two filaments, or a single larger one from a high point, to supply the place of the posterior root of the first cervical.

Asch, in his *Monograph* on the same Nerve, (p. 40,) states, "that the fibres of its posterior root are frequently intermixed on one or other side with those of the corresponding accessory. Whenever this happens, another filament of equal size quits the accessory, again to join and form the posterior root of the first cervical."

Correspondent descriptions are to be found in Bock (*on the Spinal Nerves*, p. 19,) and in Bischoff's *Monograph on the Accessory*. In the latter, the first plate represents the fact precisely as Müller supposes he was the first to discover it.

The result of careful examinations on my own part is, that occasionally the posterior root of the first cervical nerve runs for a short space in the sheath of the accessory, quitting it at a higher or lower point, without presenting any real communication between the fibres of the two nerves. I am altogether led to adopt the remark of Sabatier, (vol. iii. p. 275,) viz. "The trunk of the accessory is usually so closely connected with this nerve at its passage outwards, that it might be supposed filaments were detached from one to join the other. I have found, however, that usually, if not always, whatever might appear to be the case, there is really no continuity between them."

Hence it is clear, that the case noticed by Müller is not new; that most preceding anatomists had observed it more accurately than himself; and, lastly, that the true relation of the two nerves furnish no evidence against a doctrine resting for its support on so many facts derived from minute anatomy, pathological anatomy, and experiments on animals.—*Tiedemann's Zeitchrift*, Band v. 1835.

---

*Of the Development of the Iris.* By FR. ARNOLD.

Kieser was probably the first to notice, in the chick, that the iris, from the first, offers a complete circle, though there is a fissure in the choroid and retina. The same fact was subsequently announced in the embryo of lizards, by Emmert and Hochstetter. (*Reil's Archiv.* 10, p. 91.) The correctness of the observation, viz. the origin of the iris in the form of a complete, uninterrupted ring, has still more recently been confirmed, in addition, by Von Baer, Ammon, Seiler, and myself, in the embryo of man, in the mammiferæ, in birds, and amphibia.

*Tiedemann's Zeitchrift*, B. v. 1835.

---

